# 3D‐Printed Aerogel Metamaterials with Multiple Heterogeneous Interfaces Enables Integrated Control of Microwave Field, Acoustic Field, and Thermal Field

**DOI:** 10.1002/advs.202521820

**Published:** 2026-01-12

**Authors:** Yijie Liu, Kokila Khanal, Weimeng Chu, Sreekanth Ginnaram, Yijing Zhao, Chunlin Jia, Udeshwari Jamwal, Jiaqi Tao, Lvtong Duan, Wentao Yan, Yong Yang

**Affiliations:** ^1^ Department of Mechanical Engineering National University of Singapore Singapore Singapore; ^2^ National University of Singapore Singapore Singapore; ^3^ Department of Materials Science and Engineering National University of Singapore Singapore Singapore

**Keywords:** 3d‐printed aerogel, microwave attenuation, multiple interfaces, sound absorption, thermal insulation

## Abstract

In the new generation of communication technologies, intelligent transportation, and energy management and other emerging industrial areas, electromagnetic waves (EMW), acoustics, and thermodynamics coexist in coupled and superimposed forms. Future smart living scenarios require simultaneous mitigation of electromagnetic (EM) interference, noise pollution, and thermal flow impacts. Based on this, this study proposes constructing aerogel metamaterials to achieve integrated regulation of microwave, acoustic, and thermal field. At the microscopic scale, multiple heterogeneous interfaces are formed through the interaction between aramid nanofibers (ANF), carbon nanotube (CNT), and liquid metal (LM). At the macroscopic scale, multi‐stage resonant structures are designed to balance multiple physical fields. The aerogel metamaterial is fabricated via Direct‐Ink‐Writing (DIW) and freeze‐drying. Ultimately, the metamaterial achieves ultra‐wideband microwave attenuation (MA) from 2.65–18 GHz and TE/TM dual‐polarization robustness at oblique incidence approaching 70°; the broadband noise reduction effect in the 2800–6400 Hz; and thermal suppression where the upper surface temperature remains only one‐third of the heating field at 120°C after 30 min. The thermal conductivity of the sample is as low as 0.3649 *W*/*m* · *K*. While the density is only 35 mg/cm^3^. This study reveals the multi‐physics field cross‐scale synergistic mechanism and provides a new pathway for the integrated multi‐physics field regulation application.

## Graphical abstract

The Direct‐Ink‐Writing (DIW) 3D‐printed aramid nanofibers (ANF)/carbon nanotubes (CNT)/liquid metal (LM) aerogel metamaterial demonstrates an effective and lightweight strategy to achieve polarization insensitive & ultra‐wideband microwave absorption from 2.65‐18 GHz, broadband noise reduction effect in the 2800‐6400 Hz and thermal suppression capability spontaneously at a low density of only 35 mg/cm^3^, implying its great potential for multi‐fields involved applications.

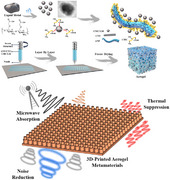

## 1 Introduction

With the advancement of the new technological revolution, multi‐physics phenomena have permeated daily life scenarios, profoundly driving social progress and industrial upgrading [1, 2, 3, 4]. In fields such as next‐generation communication technologies, intelligent transportation, green buildings, energy management, and consumer electronics, electromagnetic, acoustic, and thermal aspects no longer exist in isolation but coexist through coupling and superposition [5, 6, 7]. Future intelligent living scenarios must simultaneously address EM interference, noise pollution, and thermal shock. The coordinated regulation of multi‐physics fields directly impacts system safety, stability, energy efficiency, and living environment quality. Existing research has made progress in MA performance, noise reduction, and thermal management [8, 9, 10, 11, 12]. However, these efforts remain largely confined to optimizing individual physical fields, lacking effective strategies for integrated control within a unified system. Integrating multi‐physics regulation through cross‐scale design and manufacturing approaches holds immense potential in future technological revolutions.

As a typical ultralight porous material, aerogel is widely applied in emerging industrial fields such as microelectronics, building materials, and aerospace due to its high porosity, low density, and high performance [13, 14, 15, 16]. Owing to its open structure, aerogel can be functionally optimized by selecting different compositional systems. EM interference‐resistant aerogels can be achieved by adjusting the π‐π stacking within the 5,6‐dihydroxyindole ring tetramer framework [17]. Sound‐absorbing aerogels can be realized by crosslinking CaCl_2_ onto a scaffold composed of natural kapok fibers and sodium alginate [18]. And thermal shock‐resistant aerogels can achieve thermal insulation properties by encapsulating phase change materials within the porous framework [19]. However, single‐component aerogel systems often suffer from high brittleness, poor mechanical stability, and limited multifunctionality. They struggle to adapt to future complex, dynamic multi‐physics environments. Therefore, there is an urgent need to investigate control strategies for multicomponent aerogel systems through multiscale interface and structural design. Consequently, overcoming the limitations of traditional aerogels in multi‐physics integration.

Investigation of integrated multi‐physics regulation in aerogels can proceed from microscopic system control on one hand, and from macroscopic structural integration on the other. Metamaterial, due to their ability to artificially achieve electromagnetic parameters, acoustic impedance, and thermal conductivity unattainable in nature, have captured significant attention [20, 21, 22]. Unlike traditional materials reliant on composition and chemical modification, metamaterial enable multi‐physics integrated control through structural adjustments. 3D metamaterial introduces additional degrees of freedom along the Z–axis, expanding possibilities for multi‐physics integration. However, designing such metamaterial for multi‐physics integration is more complicated, rendering empirical design approaches increasingly rigid and unsuitable. Moreover, metamaterial designed for multi‐physics integrated control tend to involve complex, multi‐scale configurations. Fortunately, 3D printing grants greater spatial design freedom for aerogels. By inheriting the microstructural design principles of traditional aerogels, aerogel metamaterial demonstrates immense potential for multifunctional applications.

Here, we designed aerogel metamaterials capable of multi‐field integrated regulation based on a multi‐scale design principle. The multiple heterogeneous interfaces and pore structures were constructed at the microscopic level. At the macro level, a multi‐stage resonator structure is designed to achieve synergistic regulation of microwave field, acoustic field, and thermal field. Specifically, CNTs and LM are interdigitated with ANF through hydrogen bonds, π‐π interactions, and van der Waals forces, forming the structure with multiple heterogeneous interfaces. DIW and freeze‐drying are utilized to print the multi‐level porous structure. By applying Debye theory to distinguish the interaction mechanisms of each component in the multiphase system, the flat aerogel structure achieves X‐band attenuation and 186.02 kPa compressive stress. After determining the physical parameters of the aerogel, the structure of a multi‐stage resonator with central hole was designed, inspired by antenna reciprocity theory. Structural fabrication was achieved with the freedom provided by DIW. Ultimately, the metamaterial achieves ultra‐wideband MA performance from 2.65–18 GHz and TE/TM dual‐polarization robustness at oblique incidence approaching 70°; the broadband noise reduction effect in the acoustic field spanning 2800–6400 Hz; and thermal suppression where the upper surface temperature remains only one‐third of the heating field at 120°C after 30 min. While the density is only 35 mg/cm^3^. Our aerogel metamaterial with multiple heterogeneous interfaces enables integrated control of microwave field, acoustic field, and thermal field. The combination of multi‐heterogeneous‐interface aerogel, metamaterial design, and 3D printing not only provides a new technical pathway for exploring multi‐physics coupling mechanisms but also paves the way for the large‐scale fabrication and application of multifunctional aerogel‐based lightweight metamaterials.

## 2 Results and Discussion

**FIGURE 1:**
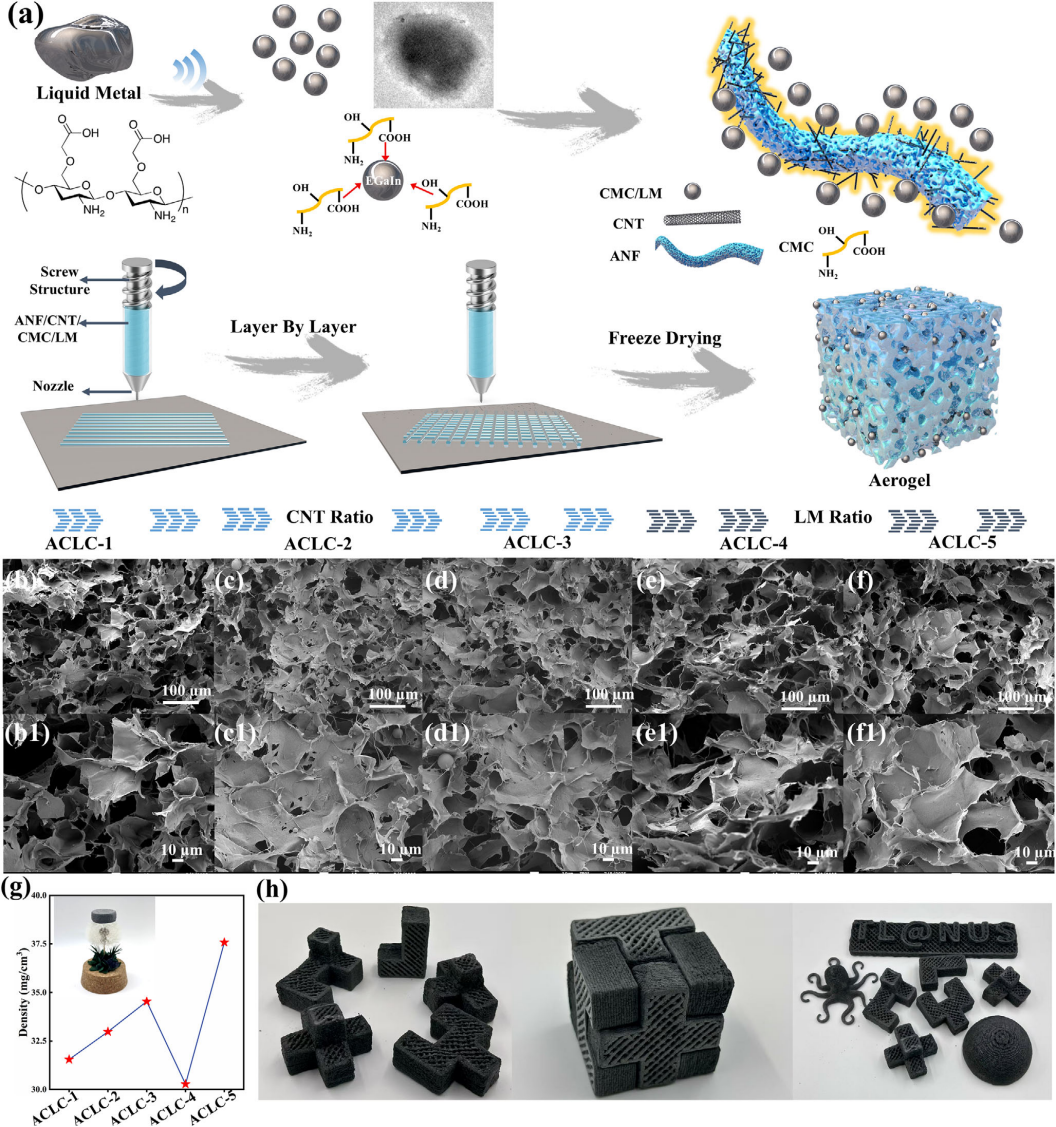
(a) Multiscale design diagram of multi‐interface aerogel. SEM of (b), b1 ACLC‐1; (c), c1 ACLC‐2; (d), d1 ACLC‐3; (e), e1 ACLC‐4 and (f), f1 ACLC‐5. (g) Density test result of the samples. (h) Optical images of aerogel printed in various shapes.

The overall multiscale design process is illustrated in Figure 1a. First, micro‐scale design is performed by introducing LM with phase‐change properties and high conductivity. However, the tendency of LM to agglomerate and undergo passivation limits its application. To enhance the compatibility of LM, carboxymethyl cellulose (CMC) with dual functional groups is used to coat it. The abundant amino and carboxyl groups in CMC chelate with metal ions in the LM, coating its surface [23]. The modified liquid metal is laminated with ANF and CNT. Hydrogen bonds, π‐π interactions, and van der Waals forces interlock these components into a multiscale structure. Subsequently, macroscale design and fabrication are performed by layer‐by‐layer printing the solution using an extrusion‐based DIW‐3D printer. This process constructs a gel structure with a complex 3D array according to the designed architecture. The gel structure undergoes pore‐making engineering via a freeze‐drying machine. Solvent sublimates after rapid freezing, leaving micrometer‐scale pores within the material. As the amount of CNT added varied, the samples were named ACLC 1–3. As the amount of LM added varied, the samples were named ACLC 3–5. The specific preparation process is described in the method and Table S1. Scanning Electron Microscope (SEM)images of aerogels with varying CNT contents are shown in Figure 1b–d. The LM particles, CNTs, and ANF form an interwoven 3D porous network structure. LM particles uniformly adhere to the ANF surface, creating a lotus‐leaf‐like structure, while CNTs bond within the network to provide structural support. As the CNT content increases, the 3Dnetwork becomes progressively more robust. The pore wall thickness and pore structure become more uniform, forming a stable multi‐scale porous framework. The aerogel ACLC‐4 without added LM particles exhibits a relatively regular pore structure (Figure 1e). In contrast, adding excessive LM particles leads to structural collapse and pore blockage (Figure 1f). This weakens the specific surface area and impedes the multiple dissipation effects of EMW. As shown in Figure 1g, the density of the tested samples exhibited a consistent trend. Notably, all samples maintained a density below 38 mg/cm^3^. The samples could even rest on a dandelion without causing deformation. This ultralight density not only enhances impedance matching with air to improve attenuation efficiency but also aligns with the demands of lightweight systems. The ACLC‐3 sample with the best structural integrity was selected for micro‐component characterization. As shown in the Figure S1, the presence of functional groups from ANF/CMC can still be detected in the infrared spectrum of ACLC‐3 [5]. In XRD testing, the amorphous carbon peak at 22° in ACLC‐3 was weakened, while the characteristic LM peak at 35° was significantly enhanced (Figure S2) [24]. This clearly demonstrates the successful composite formation of LM with ANF/CMC, which is more favorable for leveraging the high conductivity and phase‐change advantages of LM within the composite system. It is worth noting that due to the advantages of 3D printed manufacturing processes, materials can be freely printed into various shapes. As shown in Figure 1h, they can be printed not only into octopus, spherical, or logo forms, but also precisely into various cubes for assembly. This further expands the application scope and facilitates subsequent testing.

**FIGURE 2:**
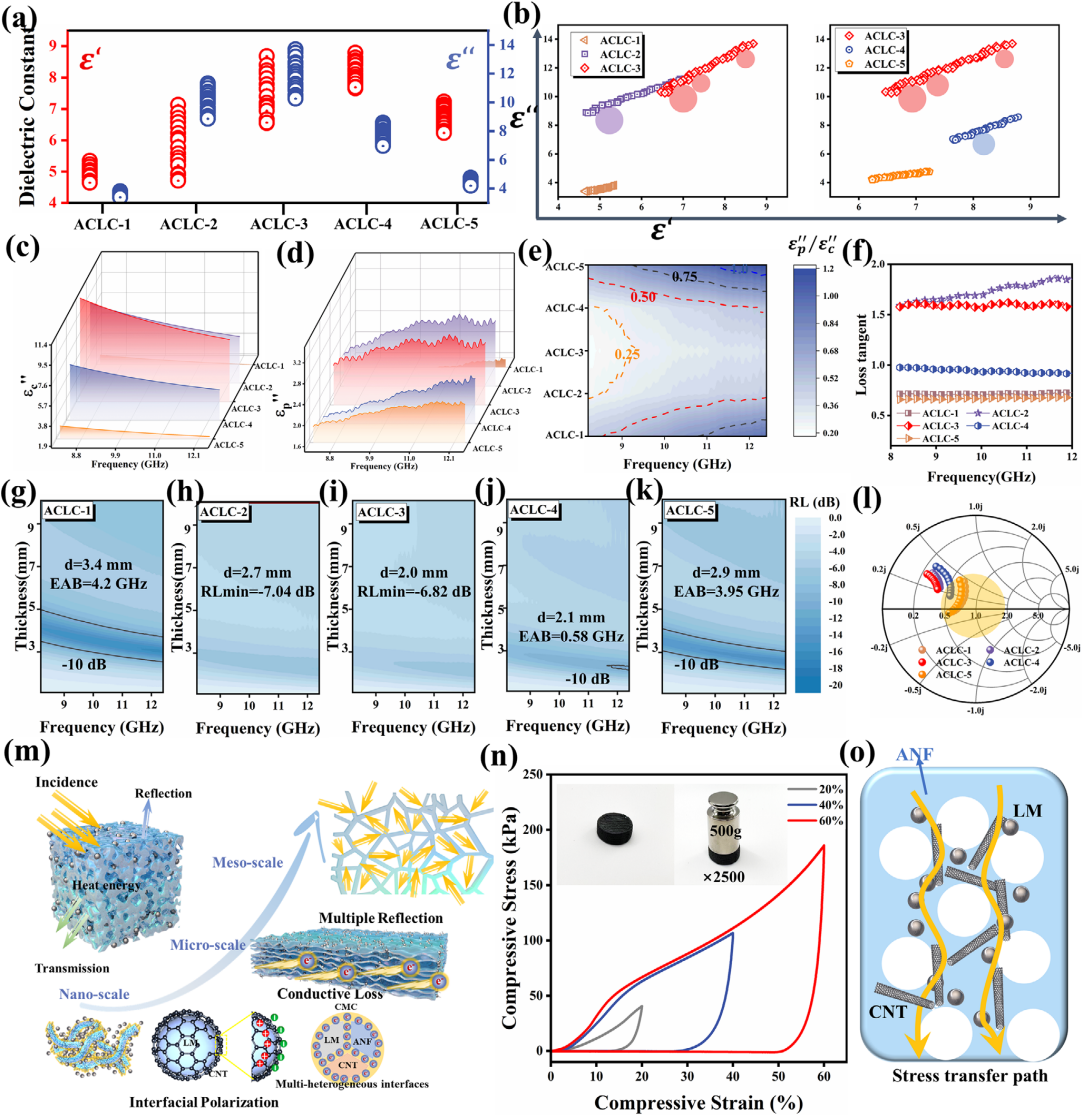
(a) Complex permittivity of the samples (real orange and imaginary blue), (b) Subdivision of the Cole‐Cole curves; Fitting results of (c) conductance loss, (d) polarization loss, and the ratio (e); (f) Loss tangent; (g–k) 2D reflection loss projection of the samples at 1–10 mm thickness, (l) Impedance‐matched Smith charts of the samples. (m) Microwave loss mechanism; (n) Compression test; (o) Multi‐scale mechanical strengthening mechanism.

Due to the significant differences in the EM properties of ANF, CMC, LM, and CNT, studying the EM parameters after multi‐phase composite formation is crucial for analyzing EMW propagation within the material. When EMW propagate into material, part of the energy increases the field energy, while another portion is converted into thermal energy and dissipated. This energy conversion process primarily stems from dielectric loss and magnetic loss. The propagation of electromagnetic waves in 3D‐printed aerogels is characterized by the complex permittivity (ε_*r*_ = ε′  − *j*ε′′) and complex permeability (μ_*r*_ = μ′  − *j*μ′′) [25]. The real part represents the capability to store energy, while the imaginary part indicates the capacity for energy dissipation. Since all samples are non‐magnetic, the focus is on the effects of variations in complex permittivity (Figure S3). The trend of complex permittivity variation across a series of samples is shown in Figure 2a. It can be observed that increasing CNT content positively influences the trend of the complex dielectric constant. At the same CNT content, the complex dielectric constant of the sample depends on a well‐ordered pore structure. Excessive LM addition disrupts the pore structure, leading to a decrease in the complex dielectric constant. The influence of CNT and LM on dielectric loss in multiphase systems can be analyzed via Debay theory. The relationship between the real and imaginary parts of the complex dielectric constant can be described by Debay theory [26]:

(1) $$\left(\varepsilon^{\prime }-\frac{\left(\varepsilon_{s}-\varepsilon_{\infty }\right)}{2}\right)+{\left(\varepsilon^{\prime \prime }\right)}^{2}={\frac{\left(\varepsilon_{s}-\varepsilon_{\infty }\right)}{2}}^{2}$$

here ε_*s*_ denotes the optical permittivity, while ε_∞_ represents the static permittivity. When polarization occurs, ε′ and ε′′ satisfy the Cole‐Cole circle. Figure 2b illustrates the Cole‐Cole curves for the two parts. In the CNT‐dominated system, the same polarization process occurs around 12 GHz. In the LM‐dominated system, the same polarization process appears at 10 GHz. Since each sample has a distinct pore structure, the ACLC‐3 sample exhibits a polarization circle around 8 GHz. Therefore, the three polarization circles observed in the ACLC‐3 sample, which exhibited the most pronounced polarization process from the low to high frequency range, are attributed to the pore structure, LM, and CNT, respectively. Polarization phenomena in multiphase systems arise from friction losses of EMW when the spatial electric dipole moment generated by asymmetric charge distribution cannot keep pace with frequency changes in alternating EM field [27]. This is caused by multiphase interface polarization, but the conduction losses imposed by CNT and LM intrinsic conductivity cannot be overlooked. Distinguishing the contributions of conductive loss ε_*c*_^′′^ and polarization loss ε_*p*_^′′^ to the total loss mechanism facilitates clearer characterization of loss contributions in multiphase systems. Incorporating conductivity into the Debay model allows further subdivision of the imaginary part of the complex dielectric constant [28]:

(2)

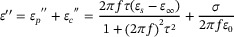

where σ is the conductivity and ε_0_ is the vacuum dielectric constant. The calculations were performed by Python least‐squares fitting of various data (Table S2). ε_*c*_^′′^ and ε_*p*_^′′^ were calculated, corresponding to parallel and series circuit models of resistance and capacitance, respectively (Figure 2c,d). It is observed that the ε_*c*_^′′^ remains strong across the entire X‐band, while the polarization loss ε_*p*_^′′^ increases with rising frequency. The calculations of the ratio ε_*p*_^′′^/ε_*c*_^′′^ reveal that conductive loss dominates throughout the entire frequency band (Figure 2e). The conductivity of CNT and LM dominates the losses. Meanwhile, the interfacial effects of the multiphase composite play a supplementary role in jointly attenuating effect. Notably, the conductive losses and polarization losses simultaneously contributed by the porous structure cannot be overlooked. Even with the highest CNT and LM loading, ACLC‐5 still exhibits low losses. Therefore, we conclude that the multiphase system of CNTs and LM must be integrated within a porous structure to maximize its effectiveness. This is further evidenced by the results of the calculations of the dielectric loss tangent (tan δ_*e*_ = ε′′/ε′ ) for the samples (Figure 2f). Due to the design of microscopic multiphase interfaces, the average tanδ_*e*_ for ACLC‐2 and ACLC‐3 samples even exceeds 1.5.

Further calculations of the MA performance through the transmission line method [29]. The attenuation characteristics of the samples at different thicknesses are shown in Figure 2g–k. Unexpectedly, the ACLC‐3 sample with higher loss exhibited poorer MA performance. Conversely, the ACLC‐1 and ACLC‐5 samples with lower loss demonstrated perfect attenuation of the X‐band at low thicknesses (<5 mm). By using the impedance matching calculations and plotting the Smith chart (Figure 2l). The yellow region indicates the impedance matching range for perfect attenuation. It can be observed that the ACLC‐1 and ACLC‐5 samples exhibit significantly superior impedance matching characteristics, thereby demonstrating better MA performance. Further insights into impedance matching can be gained through the calculations of the wave impedance coefficient ($\eta =|\sqrt{\mu /\varepsilon }|\;$) and the reflection coefficient ($R=|{\left(\frac{\sqrt{\mu /\varepsilon }-1}{\sqrt{\mu /\varepsilon }+1}\right)}^{2}|\;$) of different samples [30]. The calculations results are shown in Figure S4. It can be observed that there is a trade‐off relationship between wave impedance and reflectivity. Stronger loss characteristics imply higher reflectivity. Although the loss efficiency is improved, more EMW find it difficult to enter the material interior for attenuation. How to fully utilize the high loss characteristics at the microscopic level through macroscopic structural design is the problem we need to address next.

We first summarize the approach to designing high‐loss characteristics at the microscopic scale, which we subsequently leverage further (Figure 2m). At the nanoscale, we construct multiphase structures to enhance polarization loss. LM, CNT, ANF and CMC with differing conductivities form multiple interfaces. Differences in conductivity cause uneven charge distribution at the interface, inducing the generation of easily polarizable spatial electric dipole moments. These spatial electric dipoles rotate within alternating EM field, causing friction losses that dissipate substantial EM energy as heat. At the micrometer scale, extensive layer‐wall structures connect dispersed LM and CNT particles, forming conductive pathways that enhance dielectric losses. These structures create resistive‐like configurations in alternating electric fields, where induced currents dissipate EM energy via the Joule heating effect. Finally, mesoscale structures form multiple reflection networks to enhance reflection loss. Incident EMW undergo Rayleigh scattering within the porous layered structure, prolonging their propagation path [31]. Ultimately, the synergistic enhancement of loss modes‐polarization loss at the nanoscale, conductive loss at the micron scale, and multiple reflections at the mesoscale‐yields high‐loss characteristics. Furthermore, impedance matching can be readily tuned by adjusting internal interfaces and phase composition structures, enabling a transition from high loss with low matching to strong attenuation with high matching. Moreover, the multiscale heterogeneous interface design significantly enhances mechanical properties. The ACLC‐3 sample with optimal loss characteristics can easily withstand loads 2500 times its own weight. Young's modulus distributions at 20%, 40%, and 60% strain reached 40.61, 106.81, and 186.02 kPa, respectively (Figure 2n). At the micro‐scale, the multi‐interface pinning effect between CNT and LM not only restricts the deformation and movement of cellulose molecules to enhance self‐stability but also strengthens stress transfer pathways to improve stability under external forces. This multi‐interface assembly ultimately enhances mechanical stability, constructing a high‐strength aerogel structure. The resulting mechanical improvements enable us to design and fabricate macroscopic superstructures layer by layer, ensuring stability throughout both the printing process and subsequent applications.

The aforementioned studies reveal that multi‐interface and porous structures endow the ACLC series samples with tunable loss characteristics. However, these loss properties do not exhibit a positive correlation with their MA performance. To fully leverage the microscopic loss characteristics of the samples, macroscopic structural design is employed to guide EMW into the material interior for efficient attenuation. First, the attenuation constant is calculated [12]:

(3) $$\alpha =\frac{\sqrt{2}\pi f}{c}\sqrt{\left(\mu^{\prime \prime }\varepsilon^{\prime \prime }-\mu^{\prime }\varepsilon^{\prime }\right)+\sqrt{{\left(\mu^{\prime \prime }\varepsilon^{\prime \prime }-\mu^{\prime }\varepsilon^{\prime }\right)}^{2}+{\left(\mu^{\prime }\varepsilon^{\prime \prime }+\mu^{\prime \prime }\varepsilon^{\prime }\right)}^{2}}}$$

**FIGURE 3:**
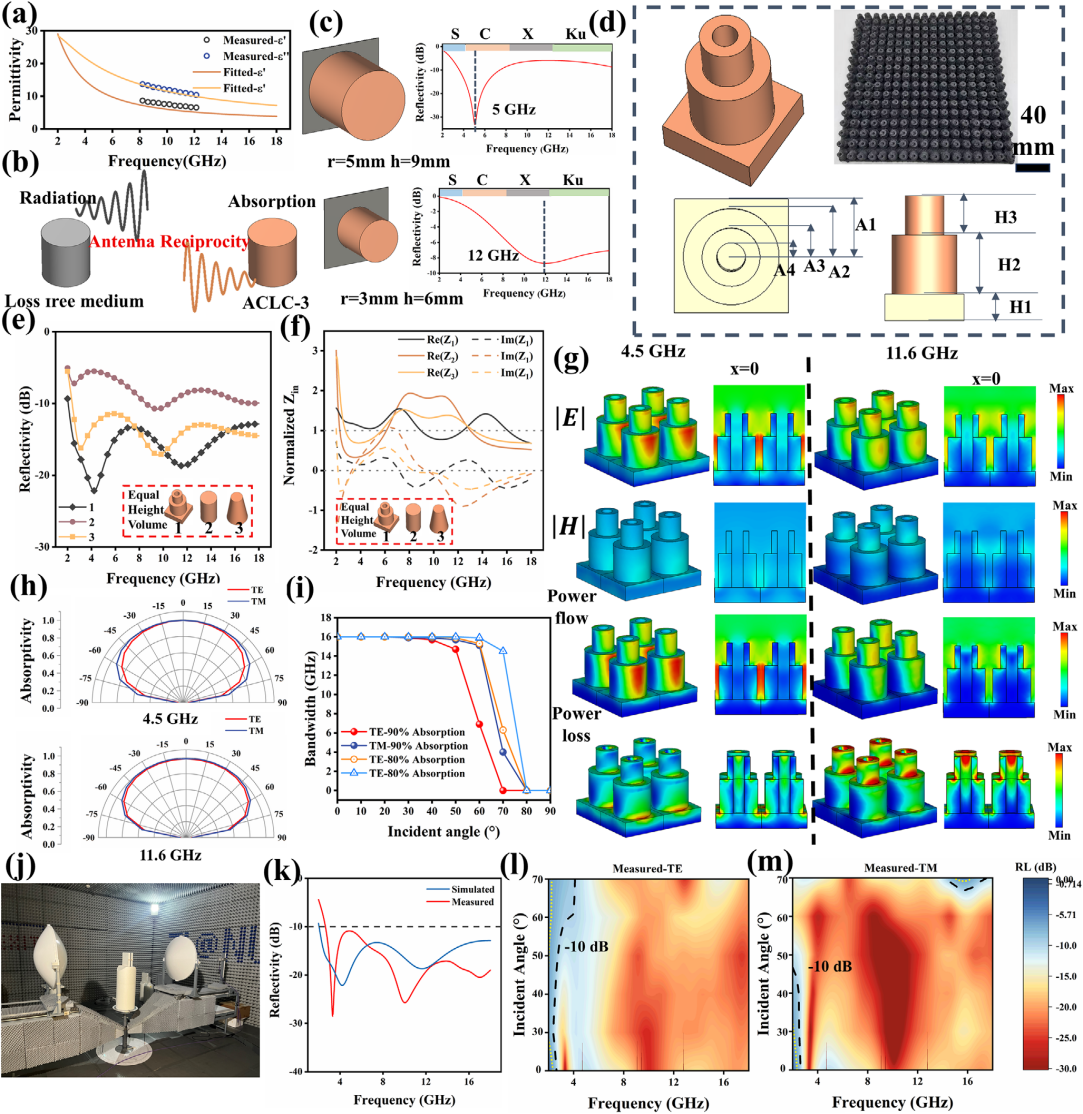
(a) Dielectric constant fitting results at 2—18 GHz. (b) Schematic diagram of metamaterial structure inspired by antenna reciprocity. (c) Resonance characteristics of cylindrical resonators of different sizes. (d) Schematic diagram of the proposed metamaterial, structural parameters, and the as‐prepared metamaterial sample. Multi‐stage resonator vs. resonant structure (e) reflectivity comparison and (f) normalized impedance at identical height and volume. (g) Distribution of electric field, magnetic field, energy flux, and power loss density in metamaterials at 4.5 and 11.6 GHz. (h) Oblique incident absorptivity of metamaterials at 4.5 and 11.6 GHz. (i) Attenuation bandwidth of metamaterial at different incident angles. (j) Free space measurement diagram. (k) Simulated and measured values of normal incidence reflectivity. Measured reflectivity heat maps of the multi‐stage resonator metamaterial at different incidence angles under (l) TE polarization and (m) TM polarization.

The ACLC‐3 sample with the strongest attenuation characteristics was selected for structural design (Figure S5). As shown in Figure 3a, since the ACLC‐3 sample exhibits dispersion characteristics dominated by ohmic loss, its complex dielectric constant from 2 to 18 GHz can be fitted using the Cole‐Cole model [32]. Traditional Dallenbach layer structures struggle to fully utilize the high loss characteristics of the material due to the competing relationship between impedance and attenuation [33]. In antenna theory, transmit‐receive antennas with identical structures radiate waves along the structure in transmission mode and capture waves of the same frequency in reception mode [34]. This phenomenon is termed antenna reciprocity. Inspired by antenna reciprocity theory, employing a high‐loss medium in the receiving structure transforms the antenna into an efficient metamaterial that effectively balances impedance and attenuation. Cylindrical dielectric resonators, chosen as prototype metamaterial designs due to their compact size, high efficiency, and versatile feeding methods, serve as the basis here. Drawing inspiration from typical cylindrical dielectric resonators and guided by antenna reciprocity, we designed a highly efficient metamaterial structure (Figure 3b).

Cylindrical resonators exhibit multiple resonance modes, among which the mode featuring simultaneous electric and magnetic field vectors along the cylindrical axis is termed the HEM_mnp_ mode. The three subscripts denote the azimuthal, radial, and axial field variations, respectively. Under grounded constraints, HEM_11δ_ and HEM_21δ_ represent the first two permissible hybrid modes within the resonator. The central resonance frequencies for different modes are as follows [35]:

(4)

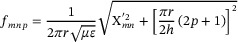

here, r denotes the radius of the cylinder, h represents height, and 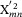 signifies the zero of the derivative of the first type Bessel function. From the above formula, it can be observed that the radius and height of the cylindrical structure are inversely proportional to the resonant frequency. Additionally, the HEM_11δ_ mode exhibits a lower resonant frequency. The aforementioned theory applies to lossless, isolated resonators. For resonator arrays with lossy media, the coupling effects between elements, high material losses, and plane‐wave excitation conditions all exert certain influences on the mode distribution. The 2–18 GHz range is typically divided into the S‐band (2–4 GHz), C‐band (4–8 GHz), X‐band (8–12 GHz), and Ku‐band (12–18 GHz). The target design aims for full‐band attenuation, but due to the wide frequency range, we optimized separately for the S/C bands and the X/Ku bands. For the low‐frequency S/C bands, the center frequency is 5 GHz. We selected 5 GHz as the resonant frequency point for the unit cell dimension calculations. The initial radius r was set to 5 mm. Using the formula, the thickness h was calculated to be 9 mm, with a period of 12 mm. For the high‐frequency X/Ku bands, the center frequency is 12 GHz. We selected 12 GHz as the resonant frequency point for the unit cell dimension calculations. The initial radius r was set to 3 mm, yielding a calculated thickness h of 6 mm and a period of 10 mm. The calculations for different resonant frequencies are shown in Figure 3c. It can be observed that, due to the broad target frequency band, the structural dimensions corresponding to different resonant frequencies exhibit significant differences. A single dielectric resonator exhibits strong narrowband attenuation but struggles to expand bandwidth. Here, we enhance broadband MA performance through multi‐stage resonator coupling. Dielectric resonators with different resonant frequencies are assembled into a multi‐stage dielectric resonator metamaterial structure, with a central cavity constructed to improve multi‐physics performance. Additionally, due to the significant conductivity difference between metal and the ACLC‐3 sample, we incorporate additional thickness to ensure comparable overall dispersion characteristics after radiation attenuation conversion. The final structural design is shown in Figure 3d. Figures S6 and S7 illustrate the influence of central cavity A4 dimensions and substrate thickness H on MA performance. Since A4 dimensions are comparable to high‐frequency resonators, it primarily affects high‐frequency behavior. Substrate thickness alters low‐frequency input impedance, thereby influencing MA performance. The optimized structural parameters are: A1 = 6 mm, A2 = 5 mm, A3 = 3 mm, A4 = 1.5 mm, H1 = 4 mm, H2 = 9 mm, and H3 = 6 mm. The optimized multi‐stage resonator metamaterial achieves an attenuation bandwidth spanning 2–18 GHz with an equivalent thickness of 5.5 mm and a density of 0.035 g/cm^3^.

Analysis of the resonant characteristics of multi‐stage resonators enables comparison of the reflectivity and normalized impedance between single‐stage resonators and conical resonators of equal volume and height. Due to the design incorporating multiple resonance points and a hollow cavity, the multi‐stage resonator exhibits the highest number of resonance points and the best impedance matching characteristics (Figure 3e,f). However, due to strong coupling effects within the multi‐stage resonator, the resonant frequencies exhibit slight deviations compared to those derived from the calculations for each stage. The two resonant points are adjusted to 4.5 and 11.6 GHz, respectively. Further analysis of the resonant characteristics at these points is accomplished with the multifield monitor. Field distribution plots for electric field, magnetic field, power flow, and loss density are shown in Figure 3g and Figure S8. It can be noticed that the overall magnetic field density is relatively weak, while the distributions of electric field, power flow, and loss density exhibit similar trends. At low frequencies, the electric field strength and power flow are primarily contributed by the first‐stage resonator. Furthermore, due to the interface changes between the substrate and the first‐stage resonator, losses are concentrated at the junction between the first‐stage resonator and the substrate. At high frequencies, the electric field strength and power flow are contributed by the second‐stage resonator. Energy dissipation occurs within the secondary resonator section. The presence of the hollow cavity further enhances loss. aligns with our design objectives. This strategy of integrating multi‐stage resonators with a hollow cavity effectively overcomes broadband attenuation limitations imposed by attenuation and impedance.

Another issue warrants particular attention. In practical applications, EMW incident at various angles exhibit random polarization. Therefore, evaluating the robustness of attenuation under different polarizations and angles is crucial. Due to the excellent matching characteristics of the multi‐stage resonator, the structure demonstrates outstanding angular stability at 4.5 GHz and 11.6 GHz when EMW incident at oblique angles in TE/TM modes (Figure 3h). Even at high frequencies, the hollow cavity design maintains 90% attenuation efficiency at a 60° oblique incidence. Furthermore, the effective attenuation bandwidth demonstrates robust stability when the incident angle varies. In the TE/TM mode, the 80% effective attenuation bandwidth begins to narrow only at 70° (Figure 3i). To accurately evaluate the MA performance of the multi‐stage resonator metamaterial, ACLC‐3 samples were fabricated into 204 mm × 204 mm × 19 mm specimens and tested using the free‐space method (Figure 3j). Results confirm that the multi‐stage resonator metamaterial ultimately achieves effective attenuation across the 2.65–18 GHz range (Figure 3k). The number of resonance points in the experimental and simulated curves matched well. However, due to uncertainties in EM parameter fitting and printing precision, the resonance peaks exhibited slight positional shifts. Surprisingly, the structure demonstrated superior robustness under different polarizations and angles compared to simulations. The multi‐stage resonator exhibited consistent effective attenuation performance for TM‐polarized modes within 70° incidence angles (Figure 3l). The attenuation stability for TE modes also approaches 60°, exceeding the anticipated attenuation robustness (Figure 3m). The attenuation stability for TE modes also approaches 60°, exceeding the anticipated attenuation robustness. This may stem from the microporous structure of the printed aerogel, which induces additional secondary reflections at larger incident angles, enhancing high‐frequency MA performance. The synergistic interaction between the microporous structure and the macroscopic multi‐stage resonator design collectively enhances the attenuation stability.

The design of multi‐stage resonant structures synergistically enhances microwave field control performance through cross‐scale coordination between micro and macro levels:

1. At the nanoscale, LM, CNT, and ANF with differing conductivities form multiple heterogeneous interfaces. This creates uneven charge distribution, generating spatial electric dipole moments that dissipate energy through friction in alternating electric fields.
2. At the microscale, extensive layer‐wall structures connect dispersed LM and CNT to establish conductive pathways. This creates a resistive‐like structure that enhances conductive losses.
3. At the mesoscale, the porous structure builds a multi‐reflection network that extends wave propagation paths, causing energy dissipation. It also effectively enhances the attenuation robustness.
4. At the macroscale, perforated multi‐stage resonant structures inspired by antenna reciprocity effectively balance the inherent trade‐off between attenuation and impedance. Hollow cavities further diversify loss modes, achieving broadband attenuation performance.

**FIGURE 4:**
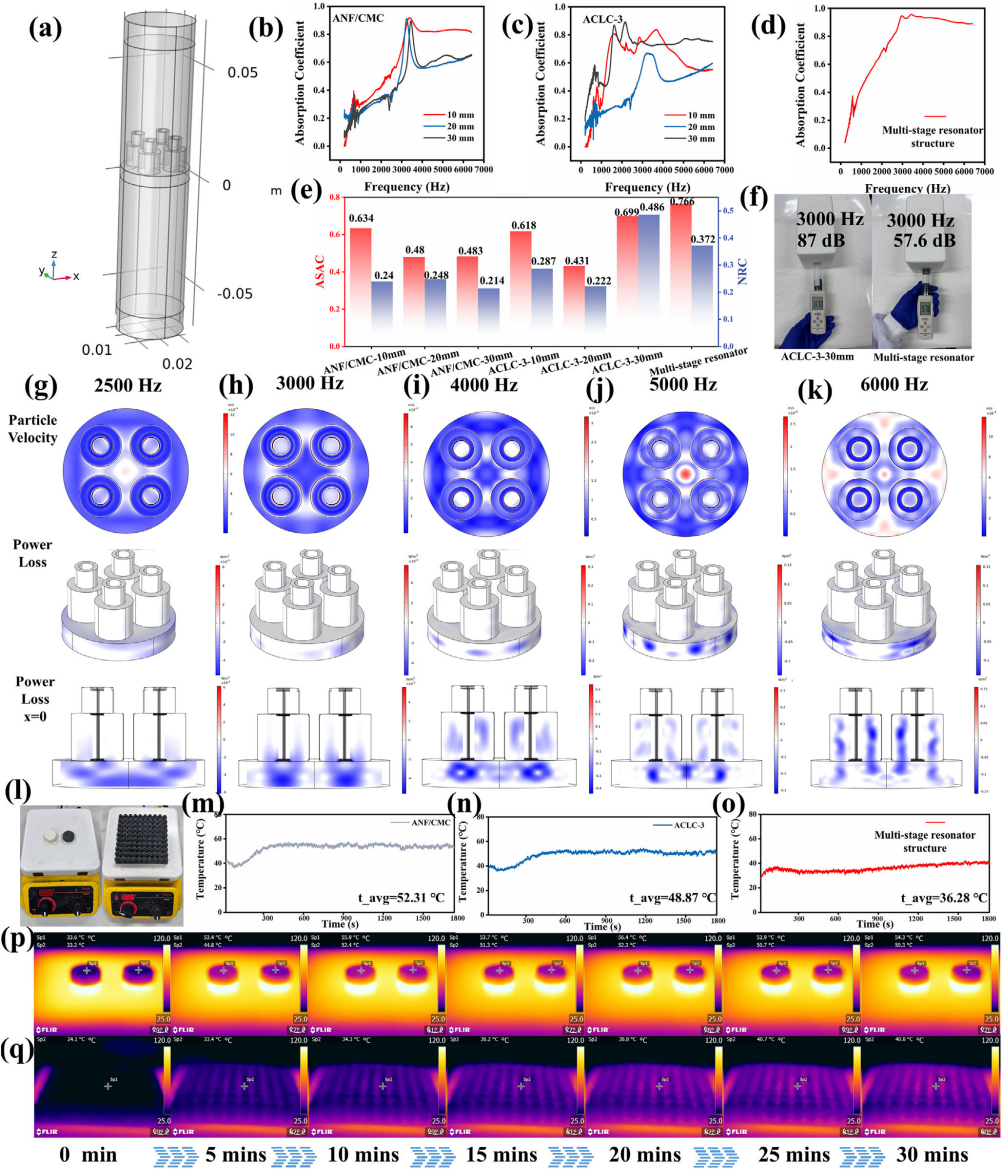
(a) Simulation model for testing a multi‐stage resonator structure in the impedance tube established using COMSOL. Sound attenuation performance testing: (b) ANF/CMC, (c) ACLC‐3, (d) multi‐stage resonator structure. (e) Comparison of ASAC and NRC of samples with different thicknesses. (f) Comparison of actual noise reduction effects between ACLC‐3 and multi‐stage resonator structure. Simulated particle velocity, power loss density distribution, and cross‐sectional distribution of multi‐stage resonator structure at different frequencies: (g) 2500 Hz, (h) 3000 Hz, (i) 4000 Hz, (j) 5000 Hz, (k) 6000 Hz. (l) Schematic diagram of thermal field control testing. Comparison of the surface temperatures of samples heated at 120°C for 30 min: (m) ANF/CMC, (n) ANF/CMC. (o) multi‐stage resonator structure. Comparison of infrared thermal imaging during 120°C heating: (p) ANF/CMC (left) and ACLC‐3 (right), (q) multi‐stage resonator structure.

The ACLC series materials feature an elaborate porous multi‐interface structure. Combined with a macro‐scale multi‐stage resonator design, it expands their application in acoustic field control. As shown in Figure 4a, the sound attenuation performance of the 29 mm diameter array structure was tested using the impedance tube method across the 250–6400 Hz frequency range. To demonstrate the necessity of both micro and macro structural design, three reference samples were selected: 3D‐printed ANF/CMC, ACLC‐3 and multi‐level resonator structures (Figure S9). The sound attenuation coefficients of 3D‐printed ANF/CMC and ACLC‐3 at different thicknesses are shown in Figure 4b,c. It can be observed that the sound attenuation performance of the samples improved with the construction of CNT/LM multi‐interfaces. It is attributed to the addition of CNT and LM enhancing the surface roughness of the aerogel network, leading to a significant increase in viscous energy dissipation and a slight decrease in thermal energy dissipation, thereby improving acoustic wave loss across the entire frequency band [36]. Furthermore, the microstructural design extends the pore size within the aerogel, improves impedance matching with air, and promotes sound wave penetration and attenuation. However, the overall sound attenuation performance remains suboptimal. Introducing the multi‐stage resonator structure into the acoustic field control reveals that the structure achieves over 90% attenuation of incident sound waves in the 2800–6400 Hz range (Figure 4d). For comparative purposes, the average sound attenuation coefficient (ASAC) and noise reduction coefficient (NRC) were evaluated for different samples. ASAC represents the average sound attenuation coefficient across the 250–6400 Hz frequency range, while NRC denotes the average sound attenuation coefficient across four 1/3‐octave bands (250, 500, 1000, and 2000 Hz) [37, 38]. The calculations indicate that the NRC and ASAC of the samples progressively improve with the multi‐interface construction strategy (Figure 4e). ACLC‐3 achieved the ASAC value of 0.699, and NRC value of 0.486 at the thickness of 30 mm. However, with the multi‐stage resonator design, the sample achieved the ASAC value of 0.766 and NRC value of 0.372 at the thickness of 19 mm. Effective attenuation is achieved at lower thickness. In addition, the actual noise reduction capabilities of the ACLC‐3–30 mm and the multi‐stage resonator were compared. As shown in Figure 4f, when a 3000 Hz sound source was placed in an enclosed space, the noise level reached 120 dB. The multi‐stage resonator demonstrated a noise reduction effect of ‐−62.4 dB, significantly outperforming the −33 dB achieved by the ACLC‐3–30 mm.

To elucidate the acoustic field regulation mechanism of multi‐stage resonators, the acoustical parameters of the material should first be calculated. Using the JCA acoustic model, the acoustic parameters of ACLC‐3 aerogel were inverted via algorithmic methods (Supporting Information). The calculations for Porosity, Resistivity, Viscous Length, Thermal Length, and Equivalent Length are 0.981, 1446787 Ns/m^2^, 121.6, 121.6, and 82.37 µm, respectively. Subsequently, the acoustic properties were simulated by modeling the impedance tube test using COMSOL. Particle velocity, surface power loss, and internal power loss at frequencies of 2500, 3000, 4000, 5000, and 6000 Hz are shown in Figure 4g–k. The particle velocity field distribution directly determines the viscous drag of air and interfacial friction effects during sound propagation. The faster the particle velocity, the more intense the relative motion between air and material, resulting in lower sound pressure. Simulation results revealed that regions of higher particle velocity within the unit cell structure primarily cluster near the hollow cavity of the multi‐stage resonator. In periodic structures, particle velocity distribution also concentrates in the central region due to reflections between different units, forming localized resonant structures. As frequency increases and wavelength decreases, the multiple reflection effects between units intensify. Multiple resonant regions emerge in the particle velocity distribution, enhancing sound attenuation at high frequencies. Simulating power loss further disclosed energy dissipation locations. At low frequencies (2500 Hz), energy was primarily dissipated in the bottom resonance mode, with multi‐stage resonance yet to activate. As frequency rose to mid‐range (3000–5000 Hz), resonance modes dominated by the hollow cavity were excited, shifting energy loss from the bottom to upper layers. Power loss was contributed simultaneously by the hollow cavity and bottom resonance modes. This multi‐mode resonance enhances sound attenuation performance. his multi‐mode resonance enhances sound attenuation performance. At high frequencies, power loss concentrates on the inner walls of the cavity. The structure exhibits an interference‐dominated power loss mechanism within the hollow cavity, sustaining its broadband sound attenuation effect.

1. The microscopic design enhances the surface roughness of the aerogel network, dramatically increasing viscous energy dissipation.
2. The construction of porous structures excites air vibrations within the pores when sound waves enter, generating viscous drag and heat exchange that cause attenuation.
3. The multi‐stage resonator design with center hole permits a stepwise evolution mechanism: local resonance‐multi‐mode resonance‐cavity interference, as frequency changes. This enhances broadband sound attenuation performance.

This multiscale‐designed structure not only effectively regulates microwave and acoustic fields but also produces synergistic effects on thermal fields. To validate the validity of the multiscale design, ANF/CMC, ACLC‐3 and multi‐stage resonator samples were placed on a heating platform at 120°C (Figure 4l). An infrared camera was used to monitor upper surface temperature changes. After 30 min of heating, the average upper surface temperatures of the ANF/CMC, ACLC‐3 and multi‐level resonator samples were 52.31 48.87, and 36.28°C, respectively (Figure 4m–o). As the multi‐scale design progressed, the multi‐stage resonator structure achieved effective suppression of the thermal field. The infrared thermal imager provided clearer visualization of this thermal suppression effect. As shown in Figure 4p, the surface temperature of the ACLC‐3 sample decreased 4°C compared to ANF/CMC with the construction of microstructures. First, the 3D‐printed aerogel possesses a porous structure, where internal air thermal resistance and pore heat dissipation weakens thermal conduction pathways. Besides, the addition of liquid metal creates an infrared reflective barrier within the aerogel. The liquid metal absorbs heat near its phase transition temperature, buffering peak temperature rises. The thermal conductivity of the ACLC‐3 sample was measured using the transient method. The results indicate that the thermal conductivity of the sample is as low as 0.3649 *W*/*m* · *K* (Table S3). This thermal radiation effect is further regulated by the macroscopically designed multi‐stage resonator structure. As shown in Figure 4o, the upper surface temperature of the sample is only 1/3 of the lower surface temperature. Due to the porous structure at the bottom, a portion of thermal radiation is initially suppressed. The thermal radiation that passes through the bottom structure undergoes multiple reflections and interference within the hollow cavity. Part of the energy becomes trapped within the cavity, resulting in significant suppression of transmitted thermal radiation. Finally, infrared thermal imaging tests were conducted on the human body at room temperature. The multi‐level resonator structure can completely suppress human thermal radiation at room temperature (25°C) (Figure S10). This further expands the application scenarios for this structure.

In a nutshell, the major reasons for the excellent thermal field suppression achieved by multi‐level resonator structures are:

1. The micro‐designed porous structure forms cross‐scale multiple barriers, disrupting thermal conduction pathways.
2. Liquid metal with multi‐interface design serves as an infrared reflective layer to reduce emission efficiency. Additionally, its phase‐change properties absorb latent heat within specific temperature ranges to provide buffering.
3. The multi‐stage resonator structure with central perforations causes multiple reflections of thermal radiation. This prolongs the residence time of radiated energy within the structure, creating complex interference patterns.

Compared to recently reported absorptive aerogels, our fabricated samples exhibit the optimal comprehensive performance in terms of effective absorption bandwidth and average noise reduction coefficient (Table S4). Furthermore, we introduce the concept of relative absorption bandwidth (relative bandwidth =$\;\frac{f_{high}-f_{low}}{f_{center}}\times 100\%$) to evaluate overall absorptive performance. The relative bandwidth of the multi‐stage resonator samples reached 148.7%. Combined with the material's inherent lightweight properties, this performance meets the demands of future multi‐physics electronic device applications.

## 3 Discussion

Overall, we designed aerogel metamaterials capable of multi‐field integrated regulation based on multi‐scale design principle. At the microscopic level, we engineered multiple heterogeneous interfaces and pore structures, constructing ANF/CNT/LM/CMC multi‐heterogeneous interfaces through hydrogen bonds, π‐π interactions, and van der Waals forces. Debye theory is employed to distinguish the interaction mechanisms among components in the multi‐phase system. Ultimately, the synergistic enhancement of loss mechanisms‐polarization loss at the nanoscale, conductive loss at the micron scale, and multiple reflections at the mesoscale‐yields high‐loss characteristics. The flat aerogel structure achieves X‐band attenuation and 186.02 kPa compressive stress. After determining the physical parameters of the aerogel, the structure of a multi‐stage resonator with central hole was designed, inspired by antenna reciprocity theory. Structural fabrication was achieved with the freedom provided by DIW. Ultimately, the metamaterial achieves ultra‐wideband MA performance from 2.65–18 GHz and TE/TM dual‐polarization robustness at oblique incidence approaching 70°; the broadband noise reduction effect in the acoustic field spanning 2800–6400 Hz; and thermal suppression where the upper surface temperature remains only one‐third of the heating field at 120°C after 30 min. While the density is only 35 mg/cm^3^. This structure not only demonstrates exceptional multi‐physics integrated control capabilities but also offers convenient manufacturing processes and design methodologies conducive to further application expansion.

## 4 Methods

### 4.1 General

The details of electromagnetic performance test, sound attenuation test and Simulation details are given in the supplementary method.

### 4.2 Chemicals

Aramid Nanofiber suspension of 3 wt.%, d∼20 nm, L∼150 µm was purchased from Jufang New Material Co., Ltd., China. Carboxymethyl cellulose (CMC, 0.6 wt.%); Carbon nanotube (0.4 wt.%); Deionized water (99%) was purchased from OCSiAI., Shenzhen, China. Sodium Carboxymethyl Cellulose powder was purchased from Sigma–Aldrich. Gallium (Ga) and Indium (In) metal were purchased from Shenyang Jiabo Metals. Co., Ltd. (China).

### 4.3 Preparation of Liquid Metal (LM) Particle

Ga and In metals with a mass ratio of 3:1 were magnetically mixed in a polytetrafluoroethylene (PTFE) container at 180°C (higher than the melting point of In metal, 156.1(C) for 30 min. The mixing process is carried out in Ar atmosphere to prevent metal oxidation. Subsequently, the liquid alloy was cooled down to room temperature and collected by syringes.

### 4.4 Preparation of ANF/CNT/LM/CMC Aerogel

First, to prepare 4wt.% of CMC solution, measure 40 g of CMC powder and 960 g of DI water in a glass beaker with a magnetic stirrer. Next, place this same beaker on a magnetic hotplate and set the setting to 100 rpm and 40°C to fully dissolve CMC powder in water. Right after, measure 5 g of LM in 95 g of 4 wt.% CMC solution in a glass beaker and place it in a sonification bath till the liquid metal is fully disperse to get LM/CMC solution. Next, measure 50 g of ANF, X (X = 4, 7, 10) g of CNT/CMC paste and Y (Y = 35, 0, 70) g LM/CMC solution and toughly mix all of these materials together in a mixing machine for 18 mins at 500 rpm. The samples were ultimately designated as ACLC‐1 (X = 4, Y = 35), ACLC‐2 (X = 7, Y = 35), ACLC‐3 (X = 10, Y = 35), ACLC‐4 (X = 10, Y = 0), and ACLC‐5 (X = 10, Y = 70). Finally, load the mixed material into the 3D printer's barrel and its ready for printing. Once, the print is ready, load the ready 3D printed sample into the freeze drier for 48 h and the outcome is a lightweight 3D printed ACLC aerogel.

## Conflicts of Interest

The authors declare no conflicts of interest.

## Supporting information




**Supporting File**: advs73762‐sup‐0001‐SuppMat.docx.

## Data Availability

All data needed to evaluate the conclusions in the paper are presented in the paper or the Supplementary Information. Additional data related to this paper may be requested from the authors.
