# Supplementary material for: 3D‐Printed Aerogel Metamaterials with Multiple Heterogeneous Interfaces Enables Integrated Control of Microwave Field, Acoustic Field, and Thermal Field
Source: Adv Sci (Weinh). 2026 Jan 12;13(10):e21820. doi: 10.1002/advs.202521820 (PMC12915125; doi:10.1002/advs.202521820)
Supplement: Supplementary file 1 — Supporting File: advs73762‐sup‐0001‐SuppMat.docx. [file ADVS-13-e21820-s001.docx]

Supplementary information

# 3D-Printed Aerogel Metamaterials with Multiple Heterogeneous Interfaces Enables Integrated Control of Microwave Field, Acoustic Field, and Thermal Field

Yijie Liu^1^, Kokila Khanal^2^, Weimeng Chu^1^, Sreekanth Ginnaram^2^, Yijing Zhao^1^, Chunlin Jia^1^, Udeshwari Jamwal^2^, Jiaqi Tao^3^, Lvtong Duan^3^, Wentao Yan^*1^, and Yong Yang ^*2^.

.

^1^ Department of Mechanical Engineering, National University of Singapore, 9 Engineering Drive 1, Singapore

^2^ National University of Singapore, 5A Engineering Drive 1, 117411, Singapore

^3^ Department of Materials Science and Engineering, National University of Singapore, Singapore 117575, Singapore

*Corresponding author & E-mail address:

Wentao Yan, mpeyanw@nus.edu.sg

Yong Yang, tslyayo@nus.edu.sg

## Methods

**Characterization:** The morphology of all as-prepared samples were recorded by a field-emission transmission electron microscope (HR-TEM, JEM-2100F) and a field-emission scanning electron microscope (FE-SEM, Hitachi, S-4800). The crystalline structure of all as-prepared nanocomposites were verified by X-ray powder diffraction (XRD) patterns (Bruker D8). The thermal conductivity testing of samples is based on the transient plane heat source principle and conducted using the Hot Disk method (DRE-III).

**3D Printing Parameters:**

**Table S1.** Detailed 3D printing parameters of all the samples.

| Process Parameters | |
| --- | --- |
| Nozzle diameter | 1.4 mm |
| Layer Height | 1.0 mm |
| Line Width | 1.0 mm |
| Wall thickness | 1.0 mm |
| Wall Line Count | 1 |
| Infill density | 45 % |
| Infill Line Width | 1.0 mm |
| Print Speed | 30.0 mm/s |
| Infill Speed | 30.0 mm/s |
| Travel Speed | 60.0 mm/s |

**Electromagnetic properties:** The electromagnetic parameters from 2 to 18 GHz were measured using a vector network analyzer (VNA, HP8722D). The sample was printed as a standard sample for X-band waveguide testing: 22.86 mm × 10.16 mm × 3 mm. Perform two-port calibration on the waveguide end face using the SOLT calibration piece prior to testing. Measure S-parameters and perform inverse calculations of complex electromagnetic parameters using the Nicolson-Ross-Weir algorithm. According to the transmission line theory, calculate the value of reflection loss at the thickness of 1-10mm:^[1]^

$R_{L}=20\log\left| \frac{Z_{in}-Z_{0}}{Z_{in}+Z_{0}} \right|$ (1)

$Z_{in}=Z_{0}\sqrt{\frac{\mu_{r}}{\varepsilon_{r}}}tanh\left[ j\frac{2\pi fd}{c}\sqrt{\varepsilon_{r}\mu_{r}} \right]$ (2)

Where $Z_{in}$ is the normalized input impedance of the material, complex permittivity $\varepsilon_{r}=\varepsilon^{'}-j\varepsilon^{''}$, complex permeability $\mu_{r}=\mu^{'}-j\mu^{''}$, $c$ is the propagation velocity of EMW in vacuum, $d$is the thickness of the material and $f$ is the microwave frequency.

**Free-Space Electromagnetic Experiments:** Experimental RL results for the multi-stage resonator metamaterial (204 mm × 204 mm × 19 mm) were measured using the free-space method. Testing was conducted in a microwave anechoic chamber with background noise below -50 dB. The metamaterial sample was clamped between two low-dielectric PMI foam supports. Electromagnetic wave transmission and reception measurements were performed using a pair of high-gain standard rectangular horn antennas operating within the 2–18 GHz frequency range. Oblique incidence tests were conducted by rotating the angle between the sample and the antennas. The incidence angles for TE and TM modes ranged from 0 to 70°

**Acoustic wave attenuation testing:** The standard impedance tube setup (BWSA SW Series impedance tubes) was adopted for acoustic wave attenuation coefficient measurements. The aerogels were cut into cylinders with diameters of 29 mm.

**Acoustic** **characterization and analysis model of aerogel.** Currently, the Delany-Bazley (DB) model and the Johnson-Chamoux-Allard (JCA) model are two main characterization and analysis models for the acoustic performance of porous materials in engineering. The DB model considers the influence of macroscopic material parameters including flow resistivity ($\sigma$) and porosity ($\varphi$), and it cannot reflect the changes in the microscopic structure on the acoustic performance. Except from the macroscopic parameters $\sigma$ and $\varphi$, the JCA model uses other three parameters, namely tortuosity ($\alpha_{\infty}$), viscous characteristic length ($\Lambda$), and thermal characteristic length ($\Lambda^{'}$), which can reflect the microscopic structure of the material. Therefore, the JCA model is more suitable to characterize and analyze the aerogel samples in this study. In the JCA model, the equivalent density ($\rho_{e\text{ff}}$) and the equivalent bulk modulus ($K_{e\text{ff}}$) can be calculated by^[2]^,

$\rho_{e\text{ff}}\left( \omega\right)=\alpha_{\infty}\rho_{0}\left( 1+\frac{\sigma\varphi}{j\alpha_{\infty}\rho_{0}\omega}\sqrt{1+\frac{4j\alpha_{\infty}^{2}\rho_{0}\omega\tau}{\sigma^{2}\varphi^{2}\Lambda^{2}}} \right)$ (3)

$K_{e\text{ff}}\left( \omega\right)=\gamma P_{0}\left( \gamma-\frac{\gamma-1}{1+\frac{8\tau}{j\omega\rho_{0}B^{2}\Lambda^{'2}}\sqrt{1+\frac{j\rho_{0}\omega B^{2}\Lambda^{'2}}{16\tau}}} \right)^{-1}$ (4)

where $j$ represents $\sqrt{-1}$; $\omega$ represents angular frequency; $\tau$ represents viscosity of air; $\gamma$ represents specific heat ratio of air; $B$ represents Prandtl number.

The viscous characteristic length and thermal characteristic length are defined as,

$\Lambda=\frac{2\int_{A} \nu_{i}^{2}\left( r \right)dV}{\int_{A} \nu_{i}^{2}\left( r_{w} \right)dA}$ (5)

$\Lambda^{'}=\frac{2\int_{V} dV}{\int_{A} dA}$ (6)

where $\nu_{i}\left( r \right)$ and $\nu_{i}\left( r_{w} \right)$ represent the flow velocity inside the pore and at the pore surface, respectively.

Based on the Eqs (3) and (4), the characteristic impedance ($Z_{c}$) and propagation constant ($k_{e\text{ff}}$) are determined as:

$Z_{c}=\sqrt{\rho_{e\text{ff}}K_{e\text{ff}}}$ (7)

$k_{e\text{ff}}=\omega\sqrt{\frac{\rho_{e\text{ff}}}{K_{e\text{ff}}}}$ (8)

The surface impedance ($Z_{s}$) is calculated by:

$Z_{s}=-jZ_{c}\cot\left( k_{e\text{ff}}t \right)$ (9)

where, $t$ represents sample thickness.

Finally, the sound attenuation coefficient ($\alpha$) is obtained as:

$\alpha=1-\left| \frac{Z_{s}-\rho_{0}c_{0}}{Z_{s}+\rho_{0}c_{0}} \right|^{2}$ (10)

**Inversion principle of aerogel.** Inversion of the five abovementioned acoustic parameters ($\varphi$, $\sigma$, $\alpha_{\infty}$, $\Lambda$ and $\Lambda^{'}$) primarily depends on iteratively fitting the sound attenuation curve obtained from impedance tube measurements with that calculated based on the JCA model described by Eqs. (3)-(10). Therefore, the inversion can actually be transformed into an optimization problem with the discrepancies between measured and evaluated sound attenuation coefficient defined as optimization objective within the target frequency, as:

$[\sigma,\alpha_{\infty},\Lambda,\Lambda^{'}]=\text{agr}\min\left( {\sum_{i=1}^{N} \left( \alpha_{e}\left( \omega_{i} \right)-\alpha_{c}\left( \omega_{i} \right) \right)}^{2} \right)$ (11)

where $N$ represents the number of samples, $\alpha_{e}\left( \omega_{i} \right)$ represents the tested attenuation coefficient at frequency $\omega_{i}$, and $\alpha_{c}\left( \omega_{i} \right)$ represents the attenuation coefficient at frequency $\omega_{i}$ calculated based on Eqs. (3)-(10).

For conventional porous materials, the following bounds apply on the design variables,

$\left\{ \begin{aligned} & 1\leq\alpha_{\infty}\leq5 \\ & 1 \mu m\leq\left( \Lambda,\Lambda^{'} \right)\leq2000 \mu m \\ & \Lambda\leq\Lambda^{'} \\ & 1000 N\cdot s\cdot m^{\text{-4}}\leq\sigma\leq5\times10^{6} N\cdot s\cdot m^{\text{-4}} \end{aligned} \right.$ (12)

The genetic algorithm is adopted to solve the optimization function in this paper, the algorithmic parameters are set as: the population size is 200, the maximum number of iterations is 300, the crossover probability is 0.95, the mutation probability is 0.01, and the target frequency is 250 Hz-6400 Hz.

## Figures and Tables


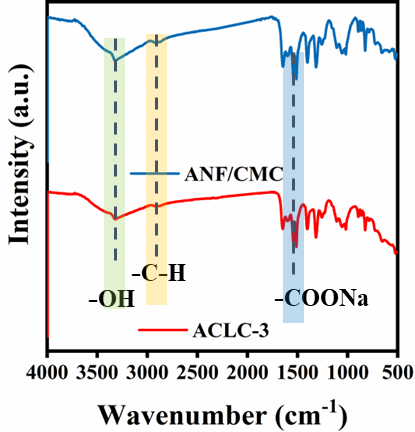


**Fig. S1:** **FTIR spectra**.


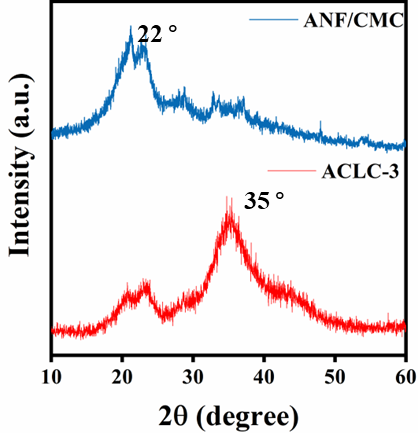


**Fig. S2:** **XRD patterns**.


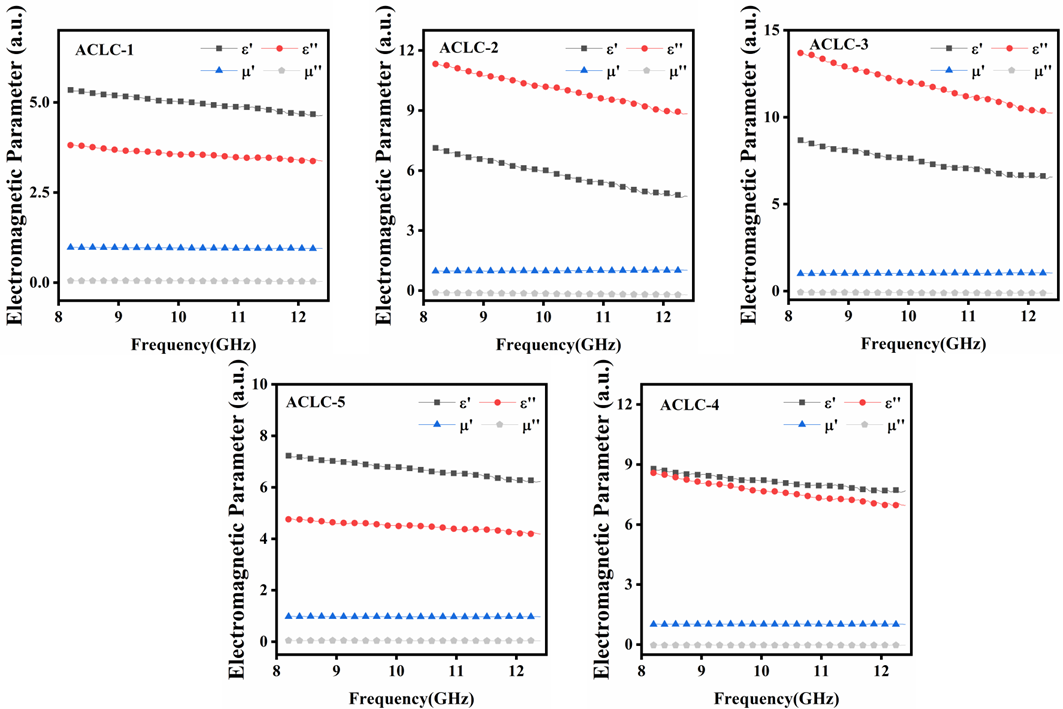


**Fig. S3** **Electromagnetic parameters of the samples**

**
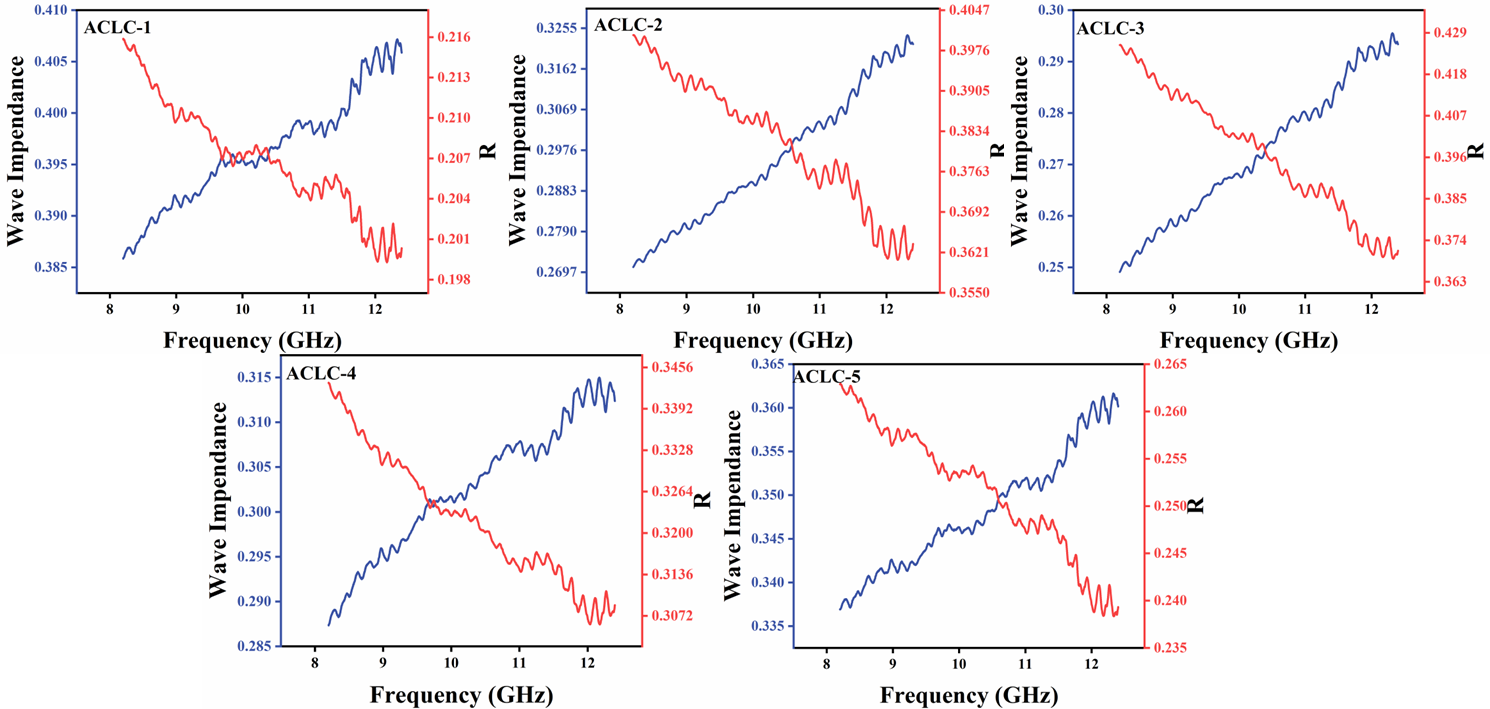
**

**Fig. S4** **Impedance and reflect of samples**





**Fig. S5** **Attenuation rate of the sample**





**Fig. S6** **Effect of substrate thickness H1 on MA performance**


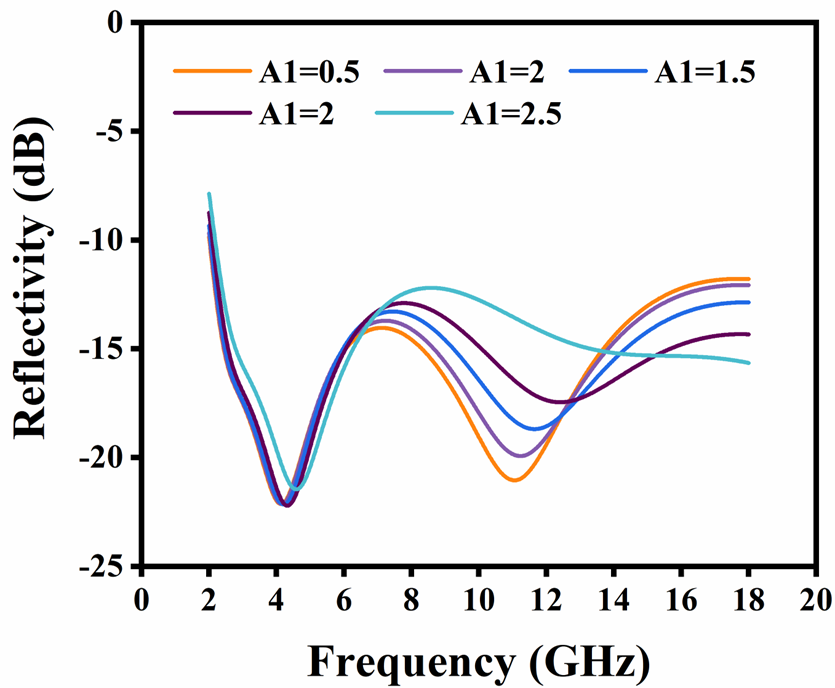


**Fig. S7** **Effect of substrate thickness A1 on MA performance**.


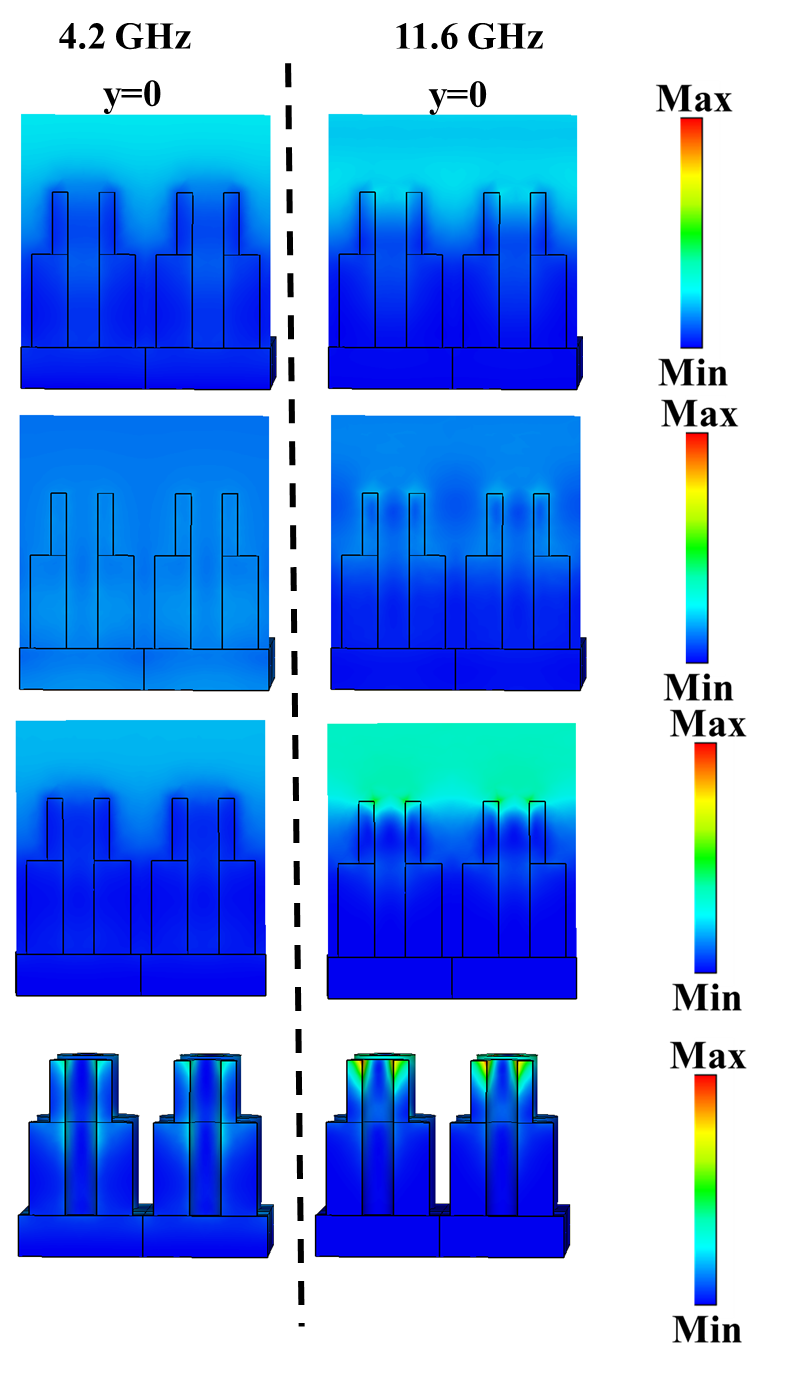


**Fig. S8** **Distribution of electric field, magnetic field, energy flux, and power loss density in metamaterials at 4.5 GHz and 11.6 GHz**.


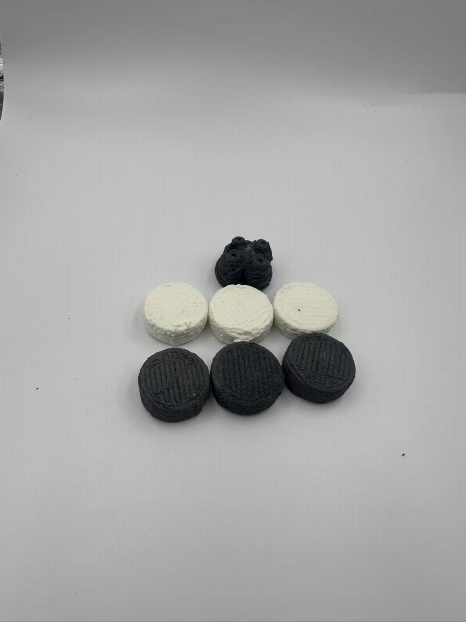


**Fig. S9** **Sound attenuation test samples**

**
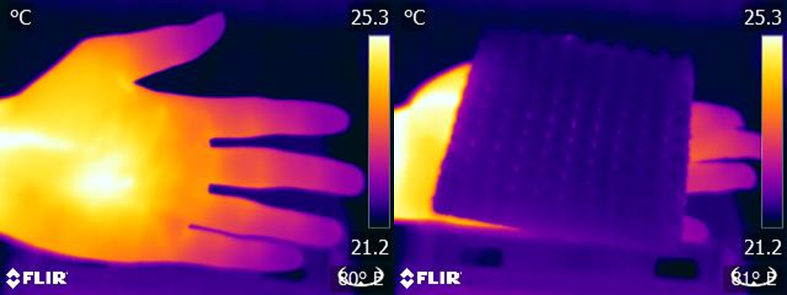
**

**Fig. S10** **The heat radiation suppression effect of the sample on human body temperature at room temperature**

**Table S2.** Detailed fitting results of all the samples.

| **Samples** | $\boldsymbol{\varepsilon}_{\boldsymbol{s}}$ | $\boldsymbol{\varepsilon}_{\boldsymbol{\infty}}$ | $\boldsymbol{\tau}_{\boldsymbol{s}}$ | $\boldsymbol{\sigma(S/m)}$ | **MSE** |
| --- | --- | --- | --- | --- | --- |
| ACLC-1 | 33.41 | 1.41 | 2.63×10^-11^ | 1.19 | 0.00019 |
| ACLC-2 | 71.31 | 1.71 | 5.28×10^-11^ | 4.21 | 0.00661 |
| ACLC-3 | 85.39 | 2.55 | 6.14×10^-11^ | 5.21 | 0.00485 |
| ACLC-4 | 87.87 | 1.01 | 3.35×10^-11^ | 3.17 | 0.00119 |
| ACLC-5 | 26.94 | 1.01 | 4.47×10^-11^ | 1.34 | 0.00081 |

**Table S3.** Thermal conductivity test results of ACLC-3 sample.

|  | **Thermal conductivity (**$\boldsymbol{W}/\boldsymbol{m\cdot K}$**)** | **Effusivity**  **(**$\boldsymbol{W\cdot}\boldsymbol{s}^{\boldsymbol{1}/\boldsymbol{2}}\boldsymbol{\cdot}\boldsymbol{m}^{\boldsymbol{-2}}\boldsymbol{\cdot}\boldsymbol{K}^{\boldsymbol{-1}}$**)** | **R Squared** | **Delta T**  **(℃)** | **Temperature (℃)** |
| --- | --- | --- | --- | --- | --- |
| 1 | 0.03627 | 44.87 | 0.9996 | 47.21 | 0.00019 |
| 2 | 0.03672 | 45.44 | 0.9996 | 47.26 | 0.00661 |
| 3 | 0.03648 | 45.14 | 0.9997 | 47.23 | 0.00485 |

The average thermal conductivity of the ACLC-3 sample is 0.3649 $W/{m\cdot K}$.

**Table S4.** Previously reported microwave absorption aerogel. ^[3]^ ^[4]^ ^[5]^ ^[6]^ ^[7]^ ^[8]^ ^[9]^

| Sample | Effective Absorption Bandwidth | Relative Bandwidth | Average Sound Attenuation Coefficient | Reference |
| --- | --- | --- | --- | --- |
| Polyvinyl Alcohol/Carbon Composite Aerogels | 12.3 GHz | 126 % | 0.55 | S3 |
| ANF/CNT/FCIP Aerogel | 4.2 GHz | 40.7 % | 0.76 | S4 |
| Cornstraw-Derived Carbon Aerogel | 5.96 GHz | 39.6% | 0.744 | S5 |
| Aramid Honeycomb Composites Filled With rGO/BC Aerogel | 13.25 GHz | 117.7 % | 0.4 | S6 |
| Nitrogen-Doped Carbon Aerogel | 7.68 GHz | 54.2 % | / | S7 |
| SiBCN/SiCnw Ceramic Aerogel | 6.4 GHz | 43.8 % | / | S8 |
| Multilayer MXene/Polyimide Aerogels | 9.41 GHz | 70.7 % | / | S9 |
| 3D Printed ANF/CNT/LM/CMC Aerogel Metamaterials | 15.35 GHz | 148.7 % | 0.766 | This work |

[1] R. C. Che, C. Y. Zhi, C. Y. Liang, X. G. Zhou, *Appl Phys Lett* **2006**, 88.

[2] D. Zong, L. Cao, X. Yin, Y. Si, S. Zhang, J. Yu, B. Ding, *Nat Commun* **2021**, 12, 6599.

[3] C. Yu, Q. Shi, H. Zhao, J. Guo, D. Lin, Y. Yao, X. Zhang, X. Jiang, *Advanced Functional Materials* **2025**, 35, 2502749.

[4] Q. Ma, J. R. Yao, C. Shen, J. T. Zhou, P. F. Wang, L. Cheng, J. L. Gao, T. J. Lu, H. Meng, *CHEMICAL ENGINEERING JOURNAL* **2024**, 500.

[5] B. Shi, Z. Xie, Y. Duan, G. Chen, Z. Li, H. Shen, Q. Chang, H. Wu, *Journal of Materials Science & Technology* **2025**, 236, 77.

[6] B. Jiang, J. X. Shang, N. Li, Y. Wang, Z. M. Hu, J. R. Yu, *COMPOSITES COMMUNICATIONS* **2025**, 58.

[7] S. Wang, X. Zhang, S. Hao, J. Qiao, Z. Wang, L. Wu, J. Liu, F. Wang, *Nano-Micro Lett* **2023**, 16, 16.

[8] J. P. Jiang, L. W. Yan, Y. J. Xue, J. T. Li, C. S. Zhang, X. X. Hu, A. R. Guo, H. Y. Du, J. C. Liu, *CHEMICAL ENGINEERING JOURNAL* **2024**, 482.

[9] X. Wang, X. Chen, Q. He, Y. Hui, C. Xu, B. Wang, F. Shan, J. Zhang, J. Shao, *Adv Mater* **2024**, 36, 2401733.
